# Statistical patterns of human mobility in emerging Bicycle Sharing Systems

**DOI:** 10.1371/journal.pone.0193795

**Published:** 2018-03-15

**Authors:** Xiangyu Chang, Jingzhou Shen, Xiaoling Lu, Shuai Huang

**Affiliations:** 1 Center of Data Science and Information Quality, Department of Information Management and E-business, Xi’an Jiaotong University, Xi’an, China; 2 Center for Applied Statistics, School of Statistics, Renmin University of China, Beijing, China; 3 Department of Industrial and Systems Engineering, University of Washington, Seattle, United States of America; Banner Alzheimer’s Institute, UNITED STATES

## Abstract

The emerging Bicycle Sharing System (BSS) provides a new social microscope that allows us to “photograph” the main aspects of the society and to create a comprehensive picture of human mobility behavior in this new medium. BSS has been deployed in many major cities around the world as a short-distance trip supplement for public transportations and private vehicles. A unique value of the bike flow data generated by these BSSs is to understand the human mobility in a short-distance trip. This understanding of the population on short-distance trip is lacking, limiting our capacity in management and operation of BSSs. Many existing operations research and management methods for BSS impose assumptions that emphasize statistical simplicity and homogeneity. Therefore, a deep understanding of the statistical patterns embedded in the bike flow data is an urgent and overriding issue to inform decision-makings for a variety of problems including traffic prediction, station placement, bike reallocation, and anomaly detection. In this paper, we aim to conduct a comprehensive analysis of the bike flow data using two large datasets collected in Chicago and Hangzhou over months. Our analysis reveals intrinsic structures of the bike flow data and regularities in both spatial and temporal scales such as a community structure and a taxonomy of the eigen-bike-flows.

## Introduction

Understanding human mobility pattern is a longstanding scientific pursuit of mankind [1–5]. Many new data resource, such as GPS trajectory [6–8] and mobile phone data [3, 9, 10], are nowadays powerful social microscopes that bring new opportunities for us to study human mobility in new mediums, allowing to “photograph” the main aspects of the society and to create a comprehensive picture of human mobility behavior. Recently, the Bicycle Sharing System (BSS) has been spreading over 1,000 cities around the world [11] as a powerful approach to improve the first/last mile connection to other transportations. Comparing with the first-generation BSS such as the White Bicycle Plan deployed in Amsterdam in 1960s, the third generation BSS highlights the integration of information technology that enables users to borrow bike from any station and return the bike to any station in a city. As nowadays the trips could be automatically recorded, this data provide a great opportunity to understand the human mobility in a short-distance trip, which could lead to better management and operation of BSSs in traffic prediction [12, 13], station placement [14–16], usage pattern analysis [17, 18], bike reallocation [19, 20], and inventory management [21, 22], all are crucial aspects to better manage BSSs to meet the population’s dynamic needs.

Generally, there are two different schools of approaches to analyze the data of BSS. One considers individual’s trip as a basic study object. For example, to provide an efficient service schedule for bike reallocation, Zhang et al. [18] considered a trip destination and duration prediction model on the individual level. Chen et al. [14] formulated the bike station placement issue as a bike trip demand prediction problem. Zhang and Yu [12] studied a trip route planning problem for individuals. Studying one trip information leads to analytical tractability, however, methodologies developed from this perspective would find limitations when considering decision-makings on the system level involving all stations and all users over time. Thus, another type of approaches aggregate individual’s trips in a time window (commonly refereed as *bike flows*, as a bike flow is the collection of all trips from an ingress station to an egress station in a time window). For instances, Li et al. [13] provided a hierarchical prediction model to predict the bike flows that will be rent from/returned to each station in a future period so that reallocation of imbalance bikes can be executed in advance. Etienne and Latifa [17] proposed a model-based clustering algorithm to classify bike stations for efficient management.

In this paper, we focus on the bike flow data as it provides system-level information. Comparing with other existing works that analyzed the bike flow data, we notice that most of the existing works largely focus on prediction using the bike flow data rather than inquiring the data for extracting system-level statistical patterns. Probably because of this, none of them aimed to conduct a delicate analysis of the variation structure in the bike flow data. On the other hand, a series of challenges arise for analyzing the bike flow data. First, it has been found that the bike flow data is very noisy, showing an intrinsic uncertainty structure in both spatial and temporal domains. This often raises up the concern of how much regularity (which then determines predictability) is embedded in the BSS data as the bikes are shared by massive users all over the city, not to mention other uncontrollable conditions such as weather, transportation infrastructure, daily transportation conditions, demographics, and geographical disparities. Second, speaking of the bike flow data as a statistical object, significant dependence has been observed among the bike flows. The dependence makes the analysis of bike flow data difficult since many classical models assume independent assumption. Third, bike flows have a high-dimensional structure. Consider a BSS with *N* bike stations, there are *O*(*N*^2^) bike flows. The high dimensionality and dependency of the bike flows present major difficulties for statistical analysis.

To overcome the aforementioned challenges, we realize that a crucial step is to decide on what spatial scale the data should be analyzed. Solid evidences are identified in our study that we should first use clustering approach to detect the community structure among stations, and then, build the analysis on these clusters rather than on individual stations. By aggregation of the bike flows in or between detected communities (called as *aggregate bike flows* (ABFs)), not only the number of bike flows is reduced into a manageable size, but also the statistical regularity embedded in the bike flow data is sharpened which can be statistically articulated by the Principal Component Analysis (PCA). Both hierarchical clustering and PCA are not model-based methods, so they do not rely on the independent assumption of bike flows. In this paper, we show that this assembly of statistical analysis pipeline could reveal interesting city-wide statistical patterns on both datasets from Chicago and Hangzhou. Note that, in this paper, we use lower-case letters, e.g., *x*, to represent scalars, bold-face lower-case letters, e.g., **x**, to represent vectors, and bold-face upper-case letters, e.g., **X**, to represent matrices.

## Results

### Data description

The two datasets used in this analysis were provided by Chicago and Hangzhou BSSs (see S1 and S2 Datasets). In the two datasets, each trip records the user ID, the trip start and end time, and the origin and destination stations. In the Chicago data, we only focus on regular subscribers of the system that form the majority of the users. Table 1 shows detailed descriptions of the two datasets.

**Table 1:** Chicago data are public and released every two quarters. Hangzhou data are private and shared by the company of Hangzhou Public Bicycle System for research purpose only.

BSS	Data set	trip	station
Chicago	2016 Q1-Q4	3,595,383	581
Hangzhou	2013 0809-1113	29,998,826	2974

**Fig 1:**
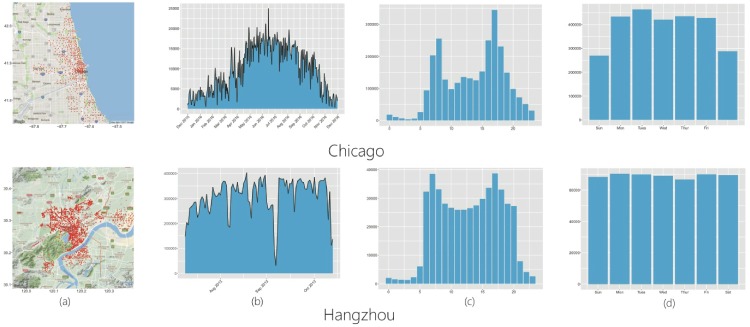
(a) Location information of the BSS stations; (b) number of trips per days; (c) average number of trips on hourly basis; and (d) average number of trips of each day in a week.

As shown in Fig 1(a), stations of both BSSs are usually located next to each other, forming a certain spatial pattern. Also, it could be seen that the number of trips per day exhibits a mix of regularity and uncertainty, as shown in Fig 1(b). For instance, the amount of trips of Hangzhou BSS is mostly stable, but could be occasionally small such as the amount of trips on the day of 7th Oct, 2013. Because this was the last day of National Holiday in China, many tourists were leaving Hangzhou city and many local customers were resting at home. This phenomenon is called short-lived property of BSS that has been reported in the literature [13, 18]. In Fig 1(c) and 1(d), we count the average number of trips on hourly basis in one day, and the average number of trips of each day in one week, respectively. It is observed that users in Chicago like using the bike in workday and rush hours, indicating that those users may have found usage of the BSS for home-workplace commutes. The average number of trips of Hangzhou BSS is relatively stable over days in one week, and have two peaks in the rush hours of one day.

### The community structure

**Fig 2:**
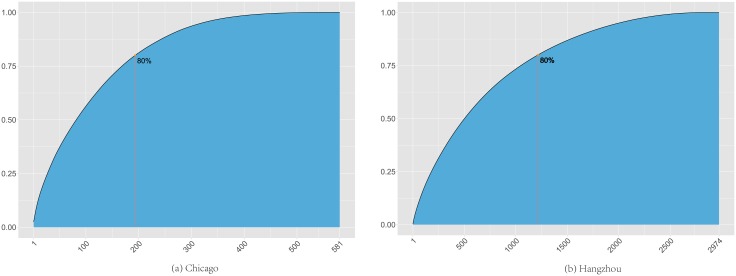
CDF of number of bike stations v.s. number of trips.

In many real-world networks, it is common that some nodes in the network would be recognized as hubs that either connect with many other nodes or contribute substantially to the network activities. It is interesting that in the bike flow data from both cities, we didn’t identify significant hub stations that can account for the amount of bike flow traffic that is significantly larger than average. To show that, we present the cumulative distribution functions (CDFs) of the number of trips in the bike stations in Fig 2. It indicates that, to account for 80% of the total trip records, it took 36% of the stations for Chicago and 42% of the stations for Hangzhou.

**Fig 3:**
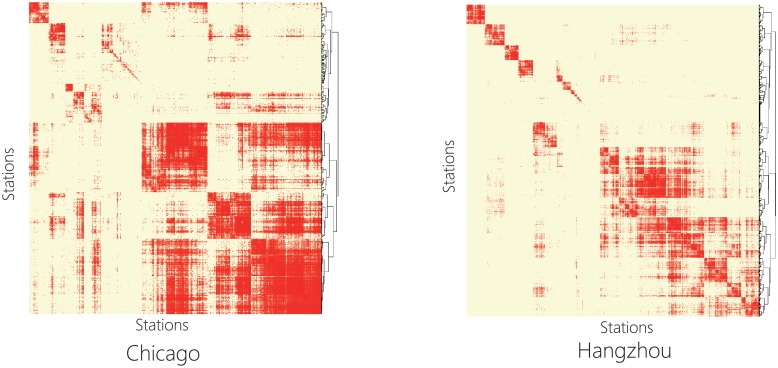
Community structures detected in both Chicago and Hanzhou bike flow data.

Although we didn’t discover significant hubs, we identified a community structure of the stations [23, 24], i.e., showing a pattern that there are many bike flows within the stations in the same cluster but less bike flows between stations in different clusters. Specifically, we used the classical hierarchical clustering method to detect the community structure. Assume that $A^{t}=\left(a_{ij}^{t}\right)\in R^{N^{2}}$ is the adjacent matrix of a BSS, where $a_{ij}^{t}(t=1,\ldots ,T$) denotes the total number of bike flows between the *i*-th and *j*-th stations in the *t*-th time epoch, *T* is the total number of time epochs, and *N* is the number of stations. Also, denote the distance at the *t*-th time epoch between the *i*-th and *j*-th stations as $s_{ij}^{t}=1/a_{ij}^{t}$. Based on the distance matrix $S^{t}=\left(s_{ij}^{t}\right)\in R^{N^{2}}$, we can construct the total distance matrix $S=\sum_{t=1}^{T}S^{t}$. By applying the classical hierarchical clustering on **S**, the bike stations are grouped 15 and 40 communities for Chicago and Hangzhou respectively in Fig 3. Note that the time interval in the paper is one hour. Thus, *T* = 24 × 366 = 8,784 for Chicago BSS and *T* = 24 × 96 = 2,304 for Hangzhou BSS, respectively.

### Aggregate bike flow (ABF) based on the community structure

Following the insight revealed by the community structure, we aggregate bike flows of the stations on the basis of clusters and conduct further statistical analysis on the aggregated bike flows (ABFs). Specifically, based on **A**^*t*^, we define that $B^{t}=\left(b_{kl}^{t}\right)\in R^{K^{2}}$ where $b_{kl}^{t}=\sum_{ij}a_{ij}^{t}I\left(i\in C_{k},j\in C_{l}\right),k,l=1,\ldots ,K$, *K* is the number of clusters, and *I*(*i* ∈ *C*_*k*_, *j* ∈ *C*_*l*_) is the indicator function that equals to one only if the *i*-th station is in the *k*-th cluster (denoted as *C*_*k*_) and the *j*-th station is in the *l*-th community (denoted as *C*_*l*_). Denote ${\tilde{x}}_{t}={\left(b_{11}^{t},\ldots ,b_{1K}^{t},b_{21}^{t},\ldots ,b_{2K}^{t},\ldots ,b_{KK}^{t}\right)}^{⊤}\in R^{K^{2}}$, and $X={\left({\tilde{x}}_{1},{\tilde{x}}_{2},\ldots ,{\tilde{x}}_{T}\right)}^{⊤}\in R^{T\times P}$ where *P* = *K*^2^. The columns **x**_*p*_, *p* = 1, …, *P* of **X** are the time series of the *p*-th bike flow in a BSS that is called *aggregate bike flows*(ABFs).

**Fig 4:**
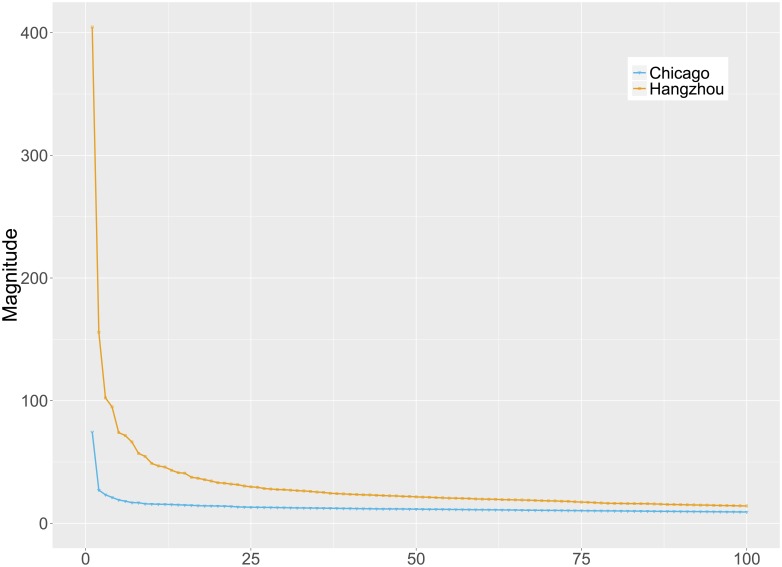
Scree plots for ABFs of Chicago and Hangzhou BSSs.

**Fig 5:**
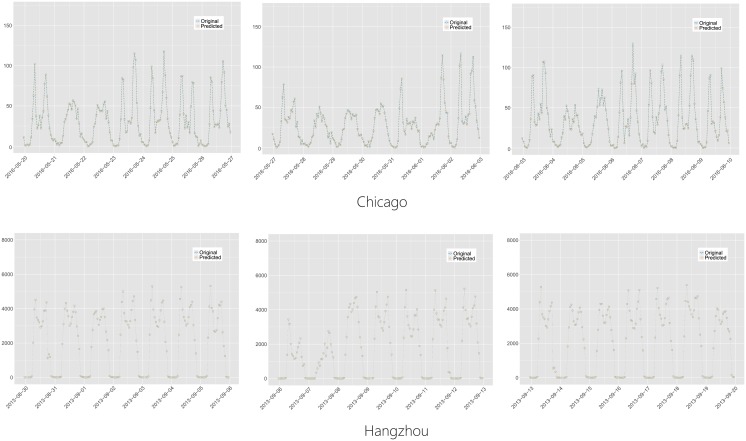
Reconstructing two ABFs with 5 principal components. For presentational simplicity, we only exhibit 3 weeks of the ABFs data.

**Fig 6:**
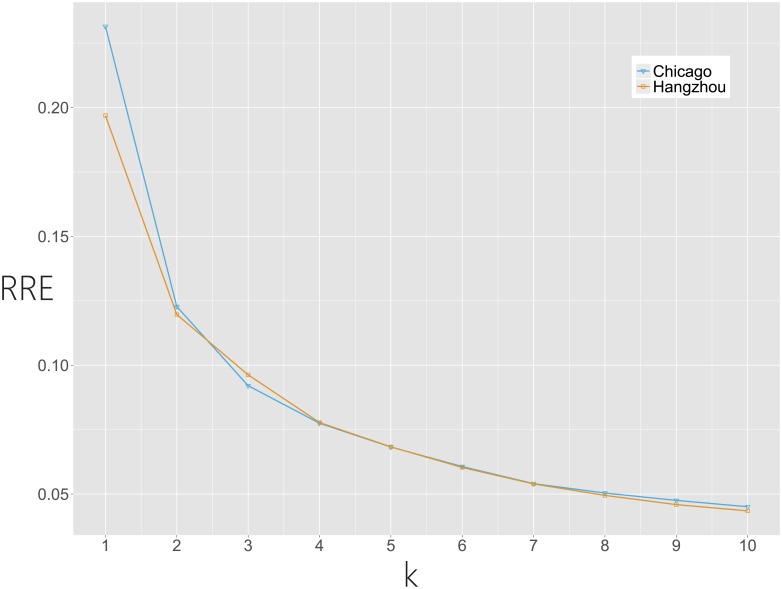
Relative reconstruction errors via the first *k* = 1, 2, …, 10 eigen-bike-flows.

By applying PCA to **X**, a low intrinsic dimensionality of the ABFs could be found in both BSS datasets, as shown in the scree plots in Fig 4. This indicates that a vast majority of the temporal variability of the ABFs is contributed by the first few eigen-bike-flows (around 5), which is much lower than the number of ABFs. As shown in Fig 5, we randomly select two ABFs and show that the two ABFs can be sufficiently approximated by the top 5 eigen-bike-flows. This observation could be generalized on all ABFs, as shown in Fig 6 that the relative reconstruction errors (RRE) via the first *k* eigen-bike-flows decrease dramatically as *k* increases, where $\text{RRE}=\|{\hat{X}}_{k}{-X\|}_{F}/{\left\|X\right\|}_{F}$, ${\left\|X\right\|}_{F}={\left(\sum_{i,j}X_{ij}^{2}\right)}^{1/2}$ and ${\hat{X}}_{k}$ is denoted by Eq 8.

### Taxonomy of the eigen-bike-flows

**Fig 7:**
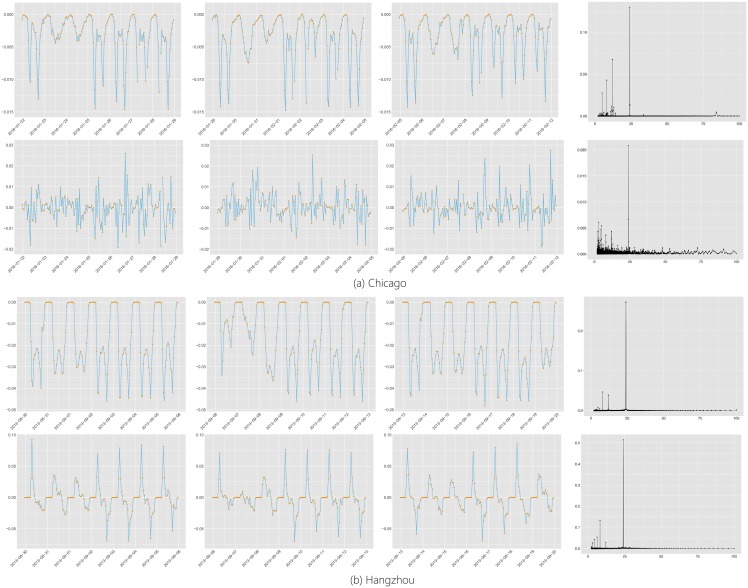
The first three columns are the examples of d-flows. The fourth column is the periodograms of d-flows. The x-axis of periodograms indicates their periods.

**Fig 8:**
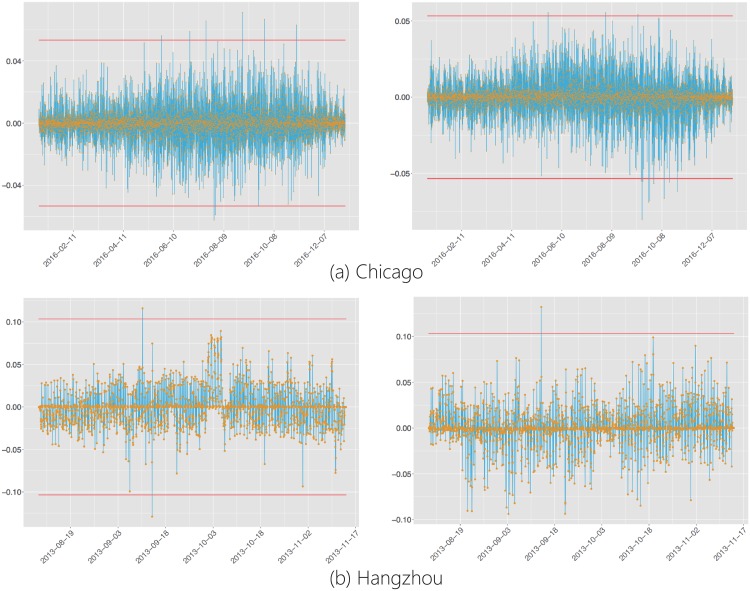
Examples of s-flows.

The aforementioned analysis of the ABF data emphasizes the central role of eigen-bike-flows in understanding the ABFs. It seems that the eigen-bike-flows can be divided into two categories: deterministic eigen-bike-flows (d-flows) and spike eigen-bike-flows (s-flows). To show this, randomly selected d-flows and s-flows from the two BSS data sets are shown in Figs 7 and 8. The d-flows in Fig 7 show periodic trends. These periodicities are reflected by the hourly (rush hour and off-peek time) and diurnal (weekday and weekend) activities. On the other hand, the s-flows in Fig 8 illustrate certain short-lived spikes, which may correspond to occasional burst of usage due to holidays or particular weather conditions.

To detect the d-flows, we conduct Fourier analysis of the eigen-bike-flows. The fourth column of Fig 7 shows that the spectrum of the selected d-flows all exhibit a significant standalone peak at twenty-four hours. To find the s-flows, the 5-sigma rule can be employed: whether there is a point whose distance from the mean exceeds 5 times standard deviations. The category of each eigen-bike-flow can be determined according to the criteria aforementioned. However, there are eigen-bike-flows who belong to more than one category. To overcome this contradiction, we define the d-flows as the eigen-bike-flows that have a significant standalone peak in the spectrum regardless the existence of the spikes.

### Statistical representational power of the eigen-bike-flows

As shown in the Method Section, each ABF can be reconstructed as a weighted sum of eigen-bike-flows (e.g., see Eq 6). Particularly, each row of the principal matrix **V** specifies the extent to which each eigen-bike-flow contributes to the corresponding ABF. Thus, we are interested to examine the rows of **V** to discern the structure of the ABFs. Particularly, for an ABF, the entries of the corresponding row of **V** whose magnitudes are remarkably larger than a threshold indicate the significant eigen-bike-flows that constitute the ABF. Here, we set the threshold as $1/\sqrt{P}$, i.e., this is because that, in an extreme situation that all the eigen-bike-flows contribute to one ABF equally, all the entries of the corresponding row of **V** will be $1/\sqrt{P}$ due to the unit norm constrain of the columns of **V**.

**Fig 9:**
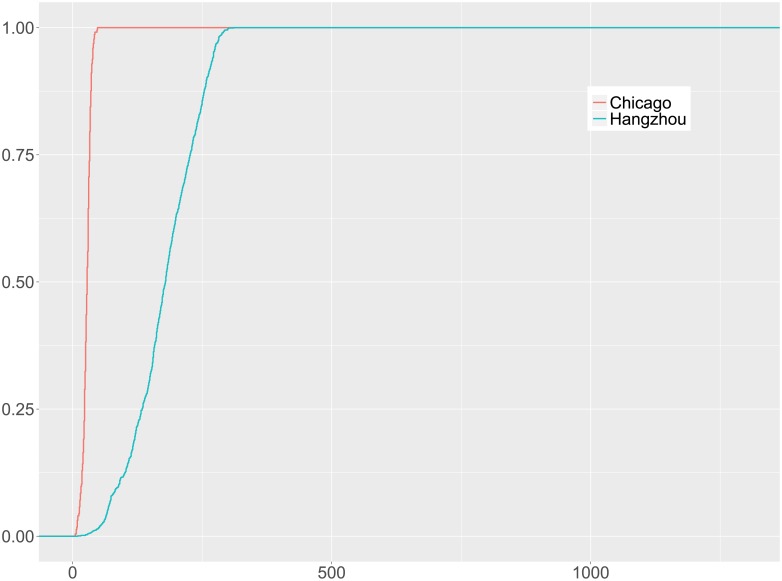
Number of eigen-bike-flows that constitute each ABF.

**Fig 10:**
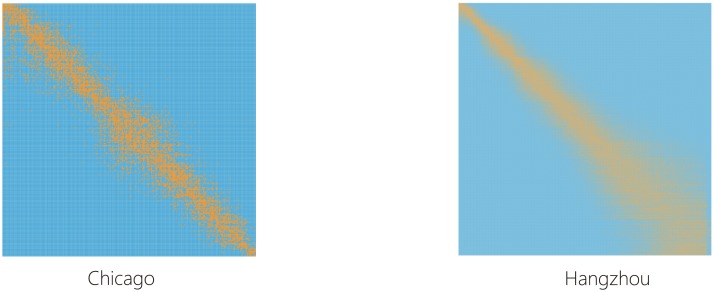
Indices of the eigen-bike-flows constituting each ABF. Note that the x-axis is the eigen-bike-flow index that are organized by convention in decreasing order of the singular values, and y-axis is ordered according to the decreasing ABF rate as well.

Furthermore, CDFs of the number of entries which exceed $1/\sqrt{P}$ in their magnitudes are shown in Fig 9. The figure indicates that overall each ABF only has a small set of constitutional eigen-bike-flows. We further show the entries of **V** whose magnitudes exceed the threshold for the two data sets in Fig 10, after the rows of **V** are sorted by the variance of their corresponding ABFs. The top rows in each plot indicate the eigen-bike-flows that are significant in forming the strongest ABFs, and the bottom rows show the significant eigen-bike-flows for the weakest ABFs. Two interesting observations can be drew. First, from the vertical direction, the elements of one ABF are clustered in a small region. Second, from the horizontal direction, the elements of the ABF with larger variance are mainly top eigen-bike-flows, while the ones with smaller variance are mainly less significant eigen-bike-flows.

### Temporal stability of the bike flow structure

**Fig 11:**
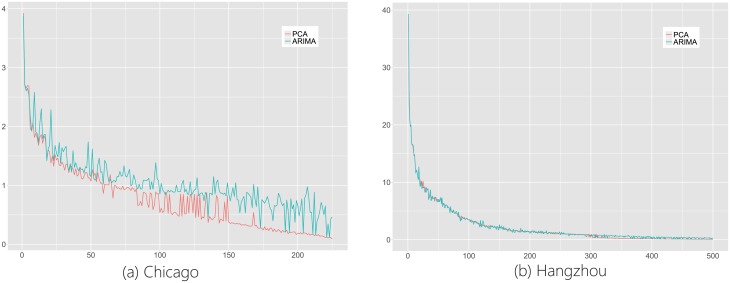
RMSE of each ABF to show the performance of ARIMA and PCA models. Note that the ABFs are ordered with decreasing variances from left to right. Furthermore, we did the Kolmogorov–Smirnov test between RMSE of ARIMA and PCA models. The p-values are all significantly less than 0.05 for Hangzhou and Chicago BSS respectively.

It is of interest to see if the bike flow structure revealed in aforementioned sections could remain stable over time. To examine its temporal stability, here, we divide the measurement matrix $X\in R^{T\times P}$ into $X_{1}\in R^{T_{1}\times P}$ and $X_{2}\in R^{T_{2}\times P}$ where *T* = *T*_1_ + *T*_2_. Then, we apply PCA on **X**_1_ and use the obtained eigen-structure to predict **X**_2_. Our rationale is that, if the eigen-structure learned from **X**_1_ is stable, then it could show significant prediction power for **X**_2_. Details of how we could leverage the eigen-structure learned from **X**_1_ to predict **X**_2_ in shown in the Section Methods. The performance of the one-step prediction of **X**_2_ is shown in Fig 11, which shows the root mean square error (RMSE) per ABF in **X**_2_, while the ABFs are ordered with decreasing variances from left to right and *T*_1_ = *T*_2_ = *T*/2. From Fig 11, it can be seen that the eigen-structure learned from **X**_1_ could lead to accurate prediction of **X**_2_. Accurately forecasting the ABFs will no doubt benefit many decision-makings for managing the BSSs such as station placement [14, 15] and bike reallocation [19, 20]. The commonly accepted approaches in bike flow forecasting consider each ABF as a time series, and then, use some time series models such as the Autoregressive Integrated Moving Average (ARIMA for short) method [25] to predict the bike flows. Here, Fig 11 also shows that the performance of the PCA-based prediction model is better than ARIMA model for most ABFs of **X**_2_.

## Discussion

The emerging bike sharing systems provide a new data source for us to understand human mobility. A unique value of this new data, comparing with existing mobility datasets such as GPS trajectory [6–8] and mobile phone data [3, 9, 10], lies on its characterization of human mobiliy in short-distance trips. Thus, as a deep understanding of the population on short-distance trip is currently lacking, we conduct a systematic analysis of the bike flow data collected in two major cities in the United States and China. Understanding the statistical characteristics of bike flow data holds great potential to develop informed decision-makings for better traffic prediction, infrastructure design such as station placement, real-time bike reallocation, and inventory management.

By analyzing the bike flow datasets from the two cities, we found statistical regularities underlying the irregular surface of the bike flow data. Our basic approach is inspired by the recognition of the spatial organizing principles of the bike flow, such that stations could be first clustered into distinct clusters. Then, by using PCA to analyze the aggregated bike flow data on the cluster level, we could identify a taxonomy of constituting eigen-flows that correspond to routine and outburst in the bike flow, i.e., (i) deterministic eigen-bike-flows, which capture the periodic trends that have been reported in previous works of other bike flow data of similar nature [13, 18]; (ii) spike eigen-bike-flows, which capture the occasional short-lived bursts [13, 18] in BSSs. Besides the interpretability of the eigen-flows, we also find out that with a small set of eigen-flows, the ABFs could be accurately reconstructed, demonstrating their statistical significance and efficiency. We further study the temporal stability of the eigen-structure by using it to predict on unseen bike flow. Thus, although irregularity could be observed from the surface of the data, regularity emerges when looking into the spatial structure embedded in data. Further, on top of the spatial structure, temporal regularity could also be detected. On what level we should interrogate the data and ask what questions seems to be a crucial precondition for us to properly understand the data and translate that understanding into better decision makings.

In contrast to some popular assumptions made in some operations research and management methods for BSS that emphasize statistical simplicity and homogeneity, our analysis reveals far more intrinsic structures, heterogeneous patterns, and statistical complexities in both spatial and temporal scales in the bike flow data. Thus, our study anticipates further development of more realistic operations research and management methods which could optimize their performances to account for the unique statistical characteristics embedded in the BSS data. On the other hand, comparing with other existing works that used the bike flow data, we notice that most of the existing works largely focus on prediction using the bike flow data rather than inquiring the data for extracting system-level statistical patterns. Probably because of this, none of them aimed to conduct a delicate analysis of the variation structure in the bike flow data. One exception [13] in these prediction works exploited the idea of first spatially clustering the stations, and then, predicting on the combined bike flows very much like the ABF in our paper. While this study showed positive evidences to backup our finding, it was motivated by gaining prediction accuracy rather than a systematic revelation of the spatial structure and temporal eigen-structure in our paper.

In summery, this paper is, to the best of our knowledge, the first attempt to comprehensively investigate the human mobility patterns in short-distance trips, characterized by their manifestation on bike sharing systems. Consistent patterns have been discovered from datasets collected in two major cities in the US and China, implying that these patterns may represent universal conditions that shape the bike flow activities in real-world. This study has limitations. First, the methods used in this study, the classic hierarchical clustering and PCA methods, reveal interesting structures but also impose limitations on the structures they could identify. Particularly, as shown in the taxonomy of the eigen-bike-flows, some d-flows contain both periodic trends and spikes. This indicates the limitations of PCA and suggests that methods that can clearly separate the underlying signals could reveal further structures in the data. Second, while PCA is useful in analyzing the ABFs, more delicate time series analysis tools or signal processing methods could be used to study the dynamics embedded in the time series data. Last but not least, the two datasets used in this study may not fully present the complexity of the BSS data in other cities. While the observations made in this study are interesting and inspiring, this study lays the foundation for further inquiries such as traffic prediction, infrastructure design such as station placement, real-time bike reallocation, and inventory management.

## Methods

### Principle component analysis

PCA, as an unsupervised statistical learning method for studying the underlying structure in complex data, has been used for coordinate transformation and dimension reduction tasks [26, 27]. It maps the original data onto a new set of axes via coordinate transformation. The new axes are referred to principal components that point to the directions with the largest variance or energy in the data. Under the assumption that the most important structure exists along the new coordinate with the largest variance, the first few principal components may well capture the concerned structure in complex data. Due to the superiority of PCA, it has been widely used in many scientific fields, such as eigenfaces for recognition [28], network traffic analysis [29] and human mobility modeling [30].

Let $X\in R^{T\times P}$ be the measurement matrix. The *p*-th column denotes the *p*-th ABF and the *t*-th row represents an instance of all the ABFs at time *t*. Deriving the principal components is to solve the eigen-decomposition problem for matrix **X**^⊤^**X**, because **X**^⊤^**X** measures the covariance between ABFs. The mathematical formulation is

$$\begin{array}{c} X^{⊤}Xv_{p}=\lambda_{p}v_{p},p=1,\ldots ,P \end{array}$$ (1)

where λ_*p*_ is the *p*-th eigenvalue corresponding to eigenvector **v**_*p*_. Since **X**^⊤^**X** is symmetric and semidefinite, its eigenvectors ${\left\{v_{p}\right\}}_{p=1}^{P}$ are orthogonal and the corresponding eigenvalues ${\left\{\lambda_{p}\right\}}_{p=1}^{P}$ are nonnegative. It is required that the eigenvectors ${\left\{v_{p}\right\}}_{p=1}^{P}$ have unit norm. Also, the eigenvalues are arranged from largest to smallest, i.e., λ_1_ ≥ λ_2_ ≥ … ≥ λ_*P*_ ≥ 0. If eigenvalues ${\left\{\lambda_{p}\right\}}_{p=1}^{P}$ have *r* nonzero values, then the rank of **X** is *r*. It is well known that ${\left\{v_{p}\right\}}_{p=1}^{r}$ are the principal components of **X**. According to (1),

$$\begin{array}{c} X^{⊤}X=V\Lambda V^{⊤}, \end{array}$$ (2)

where **V** = [**v**_1_, …, **v**_*r*_] and Λ = *diag*(λ_1_, …, λ_*r*_). Calculating principal components actually is intimately related to Singular Value Decomposition (SVD) [31]. SVD is matrix decomposition tool that can be expressed in the form of matrix multiplication as

$$\begin{array}{c} X=U\Sigma V^{⊤} \end{array}$$ (3)

where **U** = [**u**_1_, …, **u**_*r*_], $u_{i}^{⊤}u_{j}=0$ for *i* ≠ *j* and Σ = *diag*(*σ*_1_, …, *σ*_*r*_) is an *r* × *r* diagonal matrix with singular values ${\left\{\sigma_{p}\right\}}_{p=1}^{r}$ on the diagonal. Therefore,

$$\begin{array}{c} X^{⊤}X=V\Sigma^{2}V^{⊤}. \end{array}$$ (4)

Comparing with (2), we find that $\lambda_{p}=\sigma_{p}^{2}$. Furthermore, based on (3), it has

$$\begin{array}{c} u_{p}=Xv_{p}/\sigma_{p},p=1,\ldots ,r, \end{array}$$ (5)

and

$$\begin{array}{c} x_{p}=\sigma_{p}U{\left(V^{⊤}\right)}_{p},p=1,\ldots ,P, \end{array}$$ (6)

where **u**_*p*_, *p* = 1, …, *r* are vectors of size *T* and orthogonal by construction, and (**V**^⊤^)_p_ is the *p*-th row of **V**. The Eq (5) indicates that all the ABFs can be transformed into a new coordinate with weights **v**_*p*_. **u**_*p*_ captures the temporal variation common to all flows along principal axis *p*. Specifically, **u**_1_ captures the strongest temporal trend common to all ABFs, **u**_2_ captures the second strongest, and so on so forth. The Eq (6) shows that each ABF is in turn a linear combination of the eigen-bike-flows, weighted by (**V**^⊤^)_p_.

Using SVD, a low-rank approximation matrix of **X** can be constructed as follows. The approximation form is

$$\begin{array}{c} {\hat{X}}_{k}=U_{k}\Sigma_{k}V_{k}^{⊤} \end{array}$$ (7)

where ${\hat{X}}_{k}$ is an approximation of **X** with rank *k* < *r*, **U**_*k*_ and **V**_*k*_ are the first *k* columns of **U** and **V**, respectively, and Σ_*k*_ is the top-left part of Σ of size *k*. The low-rank approximation of **X** is actually a dimension reduction approach via PCA with the form

$$\begin{array}{c} {\hat{X}}_{k}=\sum_{p=1}^{k}\sigma_{p}u_{p}v_{p}^{⊤}. \end{array}$$ (8)

### Prediction of bike flow

By assuming temporal stability of the bike flow data, we could leverage the eigen-structure revealed in a previous data, denoted as **X**_1_, to predict the data in next, denoted as **X**_2_. According to SVD, $X_{1}=U_{1}\Sigma_{1}V_{1}^{⊤}$ where each column of **U**_1_ is an eigen-bike-flow and each column of **V**_1_ is a principal component of **X**_1_. We denote that $U_{1}^{d}$ as the d-flows of **X**_1_ which have periodic trends. Therefore, we can apply the ARIMA model on each column of $U_{1}^{d}$ to predict the estimated d-flows of ${\hat{X}}_{2}^{d}$. Then, based on the temporal stability assumption of the principal components, the estimation of ${\hat{X}}_{2}$ can be constructed by

$$\begin{array}{c} {\hat{X}}_{2}={\hat{U}}_{2}^{d}\Sigma^{d}{\left(V_{1}^{d}\right)}^{⊤} \end{array}$$ (9)

where Σ^*d*^ and $V_{1}^{d}$ are sub-matrices of Σ and **V**, respectively, that correspond to the d-flows in **X**_1_. The prediction performance could be evaluated by RMSE. Here, the RMSE is defined as

$$\begin{array}{c} RMSE_{i}=1/\sqrt{T_{2}}\left\|X_{2}^{i}-{\hat{X}}_{2}^{i}\right\|,i=1,\ldots ,d \end{array}$$ (10)

where *T*_2_ is the number of rows in **X**_2_, *d* is the number of d-flows, $X_{2}^{i}$ and ${\hat{X}}_{2}^{i}$ are the *i*-th columns of **X**_2_ and ${\hat{X}}_{2}$ respectively.

## Supporting information

S1 Dataset BSS data of Chicago. (CSV)

S2 Dataset BSS data of Hangzhou. (ZIP)
