# Supplementary material for: Statistical patterns of human mobility in emerging Bicycle Sharing Systems
Source: PLoS One. 2018 Mar 15;13(3):e0193795. doi: 10.1371/journal.pone.0193795 (PMC5854355; doi:10.1371/journal.pone.0193795)
Supplement: S2 Dataset — (ZIP) [file pone.0193795.s002.zip › bssdata/X_Hangzhou-README.doc]

1. Time range: 20130809-20131113.

2. Number of clusters of stations in Hangzhou: 40.

3. Each column represents the flows between two clusters, e.g., Column “0403” records the time series of flows between Cluster 04 and Cluster 03.

4. Each row represents an instance of all the flows at a certain time period, e.g., Row “20130909 11” is an instance of the flows among all the cluster pairs between 20130909 11:00:00 - 20130909 11:59:59.
